# The effects of custom-made foot orthoses on foot pain, foot function, gait function, and free-living walking activities in people with psoriatic arthritis (PsA): a pre-experimental trial

**DOI:** 10.1186/s13075-022-02808-8

**Published:** 2022-05-25

**Authors:** Roua Walha, Pierre Dagenais, Nathaly Gaudreault, Gabriel Beaudoin-Côté, Patrick Boissy

**Affiliations:** 1 Faculty of Medicine and Health Sciences, Université de Sherbrooke, Sherbrooke, QC Canada; 2 Research Center on Aging, CIUSSS de l’Estrie-CHUS, Sherbrooke, QC Canada; 3 Podiatry clinic PiedReseau, Sherbrooke, QC Canada

**Keywords:** Psoriatic arthritis, Custom foot orthoses, Foot pain, Foot function, Spatiotemporal parameters, Free-living walking activities

## Abstract

**Introduction:**

Foot involvement is a significant concern in psoriatic arthritis (PsA) as it can lead to severe levels of foot pain and disability and reduced mobility and quality of life. Previous studies have shown moderate efficacy for custom-made foot orthoses (CFO) in reducing foot pain and disability in people with rheumatoid arthritis. However, evidence on the efficacy of CFO in people with PsA is lacking.

**Objectives:**

To explore the effects of CFO on foot function, foot and lower limb pain, gait function, and free-living walking activities (FWA) in people with PsA.

**Methods:**

A pre-experimental study including twenty participants with PsA (mean age: 54.10 ± 9.06 years and disease duration: 11.53 ± 10.22 years) was carried out. All the participants received and wore CFO for 7 weeks. Foot and lower limb pain and foot function were measured before and after the intervention using the numerical rating scale (NRS) and the foot function index (FFI). Gait function was assessed by recording spatiotemporal parameters (STPs) during a 10-m walk test using an instrumented gait analysis system (Mobility Lab). Free-living walking activities (step count, free-living cadence, time spent in different ambulatory physical activities (APA)) were recorded over 7 days using an accelerometer-instrumented sock.

**Results:**

The FFI reported scores demonstrated severe baseline levels of foot pain (54.46 ± 14.58 %) and disability (46.65 ± 16.14%). Statistically and clinically significant improvements in foot pain and foot function and large effect sizes (Cohen’s effect size > 1, *p* < 0.005) were observed after the intervention period. A strong correlation (*r* = −0.64, *p* < 0.01) between the CFO wearing time and foot function was demonstrated. However, no significant changes were found for gait STP or free-living walking activities after 7 weeks of CFO use.

**Conclusion:**

Results support the clinical and biomechanical plausibility of using CFO in people with PsA to reduce pain and improve foot function. Large-scale and controlled studies are needed to confirm these findings. Moreover, a multidisciplinary approach including the prescription of exercise therapy and physiotherapy combined with CFO could be required to improve STP and promote APA in people with PsA.

**Trial registration:**

ClinicalTrials.gov, NCT05075343. Retrospectively registered on September 29, 2021

**Supplementary Information:**

The online version contains supplementary material available at 10.1186/s13075-022-02808-8.

## Background

Psoriatic arthritis (PsA) is a chronic inflammatory arthropathy and a complex disease that frequently associates skin psoriasis, musculoskeletal manifestations including axial and/or peripheral arthritis, and several comorbidities such as cardiovascular disease, diabetes, and obesity [1]. Foot and ankle problems are prevalent in people with PsA and are sometimes among the disease’s first and most important musculoskeletal manifestations [2, 3]. Several inflammatory features can be observed in the foot and ankle. These include toe dactylitis referred to as sausage-like toes [4], Achille’s tendon and plantar fascia enthesitis [4, 5], metatarsophalangeal and distal interphalangeal joint synovitis [6, 7], and tenosynovitis of the tibialis posterior, the common peroneal sheath, the long flexor tendons of flexor digitorum longus, and the flexor hallucis longus [8, 9]. All foot compartments can consequently be affected by PsA, causing pain, swelling, stiffness, tenderness, and in later stages, deformities leading to severe foot disability, reduced mobility, and quality of life [9–11].

Quantitative gait analysis, including spatiotemporal, kinematic, and kinetic parameter assessment, has been proven useful in evaluating gait function, disease progression, and/or a given intervention’s effect on patients’ mobility [12, 13]. Gait spatiotemporal parameters (STPs), in particular, are key metrics in gait function evaluation [14] and are indicators of gait adaptations in people with inflammatory arthritis [15]. Instrumented gait analysis studies in people with PsA demonstrated impairments in gait STP, including reduced cadence, gait speed, stride length, and increased double support time, indicating impaired gait function and ultimately implying difficulties in walking activities [16–18].

Walking activities, on the other hand, involve moving in diverse situations and environments (e.g., the patient’s usual environment). A significant part of daily living activities, including transport/commuting, recreation, and domestic/occupational activities, depend mostly on walking. Walking is also the most practical, accessible, prevalent form of physical activity (PA) and the most frequently prescribed activity to meet the recommendations of the international guidelines for PA [19–21]. Inherently, gait and walking are closely related. For instance, a reduced gait speed is a significant predictor of decreased daily walking activity, and it could therefore have significant impacts on daily life [22, 23]. The effects of PA and walking on health outcomes are well-established [24, 25]. It has also been reported that PA may benefit disease activity, muscle strength, fatigue, pain, and quality of life in people with PsA [26]. However, a recent systematic review demonstrated low PA levels in people with PsA which could be detrimental to PsA-associated comorbidities (i.e., cardiovascular health) and the disease per se [26]. Importantly a close relationship between foot pain and gait function deficiencies has been demonstrated [27, 28]. This suggests that interventions aiming at improving foot pain and function could eventually improve gait function and promote walking activity and overall PA in people with PsA.

PsA management is mainly based on pharmacological treatments including disease-modifying antirheumatic drugs (DMARDs), Janus kinase inhibitors (JAKi), and biological therapies which have been proven efficient on symptom control and disease activity reduction [29–31]. Remarkably, inflammation and foot pain, as well as the related disability, can still be observed in a large proportion of patients on pharmacological therapy [16, 18, 32–34]. From a physiopathological perspective, mechanical stress and trauma have been suggested as joint and soft tissue inflammation triggers in PsA [35–37]. This could partly explain the high prevalence and persistence of foot and ankle problems, considering the weight-bearing function and the continuous mechanical stress applied to the foot during daily living activities [38]. In this context, experts point out the need for targeted therapies addressing biomechanical abnormalities in the foot to reduce mechanically triggered inflammation and pain in people with PsA with persistent foot problems.

Among such therapies, custom foot orthoses (CFO) are medical devices designed to alter the magnitude and temporal patterns of the reaction forces acting on the plantar foot in order to improve foot and lower limb function and offload painful structures during weight-bearing activities [39]. CFO are common practice in patients with foot problems associated with rheumatic diseases. In people with rheumatoid arthritis (RA), a disease characterized by the high prevalence and severity of foot problems similar to those reported in PsA, the use of CFO showed positive effects on foot pain and disability [40–45]. However, to our knowledge, there are no records in the literature on the efficacy of CFO in people with PsA. In addition, despite the similarities in foot problems, RA and PsA are different entities with different clinical presentations making the direct extension of results from RA to PsA inadequate [46]. Therefore, the primary objective of this study is to explore the effects of using CFO for a 7-week period on foot function in people with PsA. The secondary objectives are to explore the effects of CFO on foot pain, the relationship between the CFO wearing time and foot pain and function, and the effects of CFO on gait function (STP) and free-living walking activities (FWA) in people with PsA.

## Methods

### Study design and participants

A pre-experimental study with pre-test/post-test design was conducted. Potential participants were referred to the research team from the rheumatology out-patient clinics at the *Université de Sherbrooke Hotel Dieu* University Hospital (CHUS). The first author (RW) screened the potential participants for eligibility via phone interviews. Twenty-two participants with PsA met the inclusion criteria and were then recruited. Inclusion criteria were being between 20 and 70 years of age, having a confirmed diagnosis of PsA by a trained rheumatologist, and having moderate to severe and recurrent foot pain and stable medication over 3 months preceding the recruitment. Patients with diabetes, neurological disease, or any musculoskeletal disease that could impact the normal gait pattern who received an intra-articular corticosteroid injection or any conservative foot treatment such as foot orthoses/footwear intervention within the past 3 months were excluded. The study was approved by the CIUSSS de l'Estrie-CHUS Ethics Board, and all the participants gave their informed consent before data collection.

### Data collection procedure

On a first data collection session (T1) at Sherbrooke’s Research Center on Aging, demographic and baseline characteristics were assessed. Then, foot and lower limb pain, global pain, and foot function were measured using self-reported questionnaires. In this same session, all the participants performed three 10-m walking trials at their self-selected speed. Participants were then examined by a trained podiatrist who performed foot casts and designed the CFO. At the CFO delivery visit (T2), the participants’ foot and lower limb pain and foot function were evaluated for a second time. This second pre-intervention measurement was planned considering the T1–T2 delay and the fluctuating nature of pain in PsA. CFO were then dispensed and adjusted if necessary to improve fit and comfort. The participants were directed to wear the CFO for 7 weeks and they were provided with a diary to record daily CFO wearing time, weekly foot pain intensity, and any changes in medication that occurred during the intervention period. At a final follow-up visit scheduled at the end of the 7-week period (T7), foot and lower limb pain and foot function were re-assessed and the instrumented 10-m walking trials were repeated. Free walking activities (FWA) were recorded over seven consecutive days before the CFO first use and after the 7-week intervention period using an instrumented sock (including an IMU at the ankle) that the participants wore during waking hours.

### Intervention

Functional foot orthoses were custom-made for each participant. The CFO were designed by the same experienced podiatrist (GBC) based on a detailed clinical and biomechanical assessment. Foot scans were obtained with the Occipital Structure Sensor 3D scanner (Occipital Inc), the patient in a prone position while the subtalar joint was held in a neutral position. The obtained scan was edited and smoothed using the MSoft software (Techmed3d Inc, Levis, QC, Canada). Three-quarter length CFO were 3D printed in a rigid material (Nylon) using a MultiJet Fusion 3D printer (HP, Palo Alto, CA, USA). The thickness of the CFO ranged between 2 and 3.4 mm depending on the participants’ medial arch weight and height. Most of the orthoses included a medial arch support, a heel stabilizer, and a metatarsal pad. The degrees and types of corrections added to the CFO were determined specifically for each participant based on the clinical examination and were adjusted at the CFO delivery according to each patient’s comfort and tolerance. The participants were taught to wear the CFO progressively during the first 2 weeks to allow for the lower limbs’ muscles and structures to adjust to the CFO, and to wear the orthoses for the next 5 weeks, 7 days a week as often as they could. To document adherence to the CFO, all the participants completed a diary to report the daily wearing time in hours. In addition, the participants were advised to wear the CFO with adapted shoes after the general characteristics (e.g., heel height, malleability of the sole, etc.) of such shoes were explained.

### Independent variables

#### Sociodemographic, baseline characteristics, and control variables

Sex, age, body mass index, professional occupation, and previous history of foot injuries were assessed using a self-reported questionnaire. Disease duration and C-reactive protein (CRP) levels at baseline were obtained from the patients’ medical records. Foot type, foot deformities, and foot pain sites were obtained from the podiatrist’s clinical examination records. Changes in medication during the CFO intervention period were recorded by the participants in the diaries provided to them.

### Outcome measures

#### Foot function

Foot function was measured using the foot function index (FFI). The FFI is a valid, reliable, and responsive self-reported questionnaire widely used in previous studies to evaluate CFO effects on foot function [47, 48]. The FFI is composed of 23 items divided into three subscales measuring foot pain (FFI-P), foot disability (FFI-D), and foot-related activity limitation (FFI-AL). Each item of the FFI is recorded on a zero-to-ten numeric rating scale (NRS) allowing for subscale and total score calculation. Each subscale score is calculated as the sum of all its items divided by its maximum possible total [47]. The total score is obtained as the sum of all subscales’ final percentages divided by the total number of subscales. The values reported are presented as percentages ranging between 0 and 100%, with higher values indicating greater pain, disability, and activity limitation. The minimal clinically important difference (MCID) for the FFI total score was found to be 7 points in patients with plantar fasciitis [49].

#### Foot pain

Foot pain intensity was measured using a zero-to-ten NRS which is a valid, reliable, and responsive tool for pain intensity assessment [50]. The MCID reported for the NRS in people with chronic pain is equal to 2 points [51]. Participants were asked to circle the number between 0 and 10 that matched better their average foot pain intensity in the 7 days preceding the data collection. Foot pain was assessed more in detail (e.g., pain walking with foot orthoses, pain walking with shoes, pain at the end of the day, etc.) with the FFI pain subscale. Moreover, to monitor the evolution of foot pain intensity during the intervention period, the patients were asked to report at the end of each week their perceived foot pain in a diary using zero-to-ten NRS. Foot pain and foot function were measured at three time points namely, at first data collection (T1), at CFO delivery (T2), and after the 7-week intervention period (T7). As there were no statistical differences in the FFI and NRS scores between T1 and T2, the average of these two time points was used as a baseline measure while the last time point (T7) represented the final measure.

#### CFO wearing time

The daily CFO wearing time was recorded in the participants’ diaries and it is reported as the average reported time per week.

#### Global and lower limb pain

Global pain and pain at the knee, hip, and lower back pain were measured at T1, T2, and T7 using the NRS (0, no pain, to 10, worst imaginable pain). Similarly to foot pain and foot function, data obtained during the first two time points were used as baseline measures.

#### Gait function

Gait function was assessed using an instrumented gait analysis system. Gait spatiotemporal parameters (STPs) including cadence, gait cycle duration, gait speed, stride length, double support, swing time, foot strike angle, and stride time variability were recorded using the Mobility Lab system (APDM Wearable Technologies) during the 10-m walk test (10MWT). Mobility Lab is a research-grade system proven to accurately and reliably estimate STP [52–54]. Mobility Lab uses a set of six OPAL inertial measurement units (IMUs) and a software that allows for automated and easy extraction of STP. All the participants performed three trials of the 10MWT. To do so, they walked over a 14-m straight walkway with the Mobility Lab’s IMUs fixed with elastics straps on the chest, the lower back, both wrists, and feet. The 10MWT trials were performed at the participants’ comfortable speed and the average of the three trials was calculated.

#### Free-living walking activities (FWA)

FWA including step count, free-living cadence, and time spent in ambulatory physical activity (APA) intensity-based categories were measured using an instrumented sock (Sensoria Inc, Redmond, WA, USA) including a 9-axis IMU positioned at the ankle. The instrumented sock connects automatically, without any manipulation needed from the participants, via Bluetooth to a smartwatch (Apple Watch, series 3) where the raw inertial measures of motion (3D accelerometer) are stored. The data are then transferred to a computer where they are processed to extract walking activities’ specific outcomes (see Additional file 1 for signal processing and step identification description). Step count was assessed as the total number of steps per day. Furthermore, to describe the step accumulation pattern throughout the day, the number of steps was assessed for each of the following active event categories based on the number of consecutive steps within each category: 0 to 20 steps, 20 to 60 steps, 60 to 120 steps, 120 to 300 steps, 300 to 600 steps, and more than 600 steps. Free-living cadence included cadence averaged for total wearing time (mean cadence/day). Moreover, as cadence may vary depending on the number of consecutive steps performed, it was also assessed for each of the above active event categories. APA intensity-based categories were determined using cadence data. They included stepping activities (0 to 59 steps/min), slow walking (60 to 79 step/min), medium walking (80 to 99 step/min), moderate intensity APA (MAPA) (100 to 119 step/min), and vigorous APA (VAPA) ( > 120 step/min), as previously defined [55, 56]. The time spent in each of the last three categories was calculated only for purposeful walking (i.e., events composed of more than 60 steps). Free-living walking activities were assessed over a period of 7 consecutive days before and after the intervention. To do so, the participants were instructed to wear the instrumented sock during waking hours while they perform daily activities. Only valid days defined as days with at least 8 h of recordings and only valid participants with a minimum of one valid day were included in the analyses. Adherence to the instrumented sock use was tested by the system itself and by asking the participants to record the daily wearing time in a diary. To avoid influencing the participant’s physical activity, they were only told that the study would assess the effects of CFO on foot pain, foot function, and gait STP.

### Sample size and statistical analysis

Given the exploratory nature of this study, the sample size was calculated assuming a large effect size (0.8) for the pre-post differences in the FFI total score. Using a two-tailed paired *T*-test, a significance level *α* of 5%, a power of 90%, and assuming a 15% dropout rate, a total sample size of 22 participants was required. The Shapiro-Wilk test was used to examine data distribution and parametric and nonparametric statistical tests were used depending on data distribution. Paired *t*-tests (or the Wilcoxon signed-rank test) were used to assess the differences in foot pain, foot function, and global and lower limb pain between baseline and T7. Cohen’s effect size was calculated to quantify the magnitude of these differences. A one-way repeated measures ANOVA was conducted to determine whether there was a statistically significant difference in weekly recorded foot pain intensity and CFO wearing time over the 7-week intervention period. Spearman correlation coefficients were calculated to assess the relationships between the CFO wearing time, foot pain, and foot function at T7. As a 2-week adaptation period was required, correlation analyses were performed using the average CFO wearing time per week calculated for the last 5 weeks of the intervention period. Correlation coefficients were considered weak, moderate, and strong for values between 0.1 and 0.3, 0.3 and 0.5, and > 0.5, respectively [57]. The pre-post differences in gait function and free-living walking activities were assessed using the Wilcoxon signed-rank test. The significance level was set at 0.05. The statistical analyses were performed using SPSS version 26 (IBM statistics Corporation, Armonk, NY).

## Results

The participant flow throughout the study is presented in Additional file 2. Twenty-seven participants were initially screened, five were excluded as two did not meet inclusion criteria and three declined to participate. Twenty-two participants met the inclusion criteria and participated in the study. Of these, one participant was excluded after the podiatry examination because the CFO was considered not clinically appropriate. Another patient was excluded because of an acute arthritis flare during follow-up. From September 2019 to August 2021, twenty participants completed the study and were included in the analyses.

### Demographic and baseline clinical characteristics

Demographic and baseline clinical characteristics are presented in Table 1. The study sample was composed of 5 males and 15 females with a mean age of 54.10 ± 9.06 years, a mean BMI of 29.3 ± 4.5 kg/m^2^, and a mean disease duration of 11.53 ± 10.22 years. The CRP baseline levels were within the normative ranges for 75% of the patients. Two participants recorded changes in medication during the intervention period (Table 1). The first participant started taking NSAIDs at the end of the fourth week for generalized body pain, and the second received a new biological therapy starting at week 4.

**Table 1:** Demographic and baseline characteristics

Variables	Mean ± SD/number (%)
**Age (years)**	54.10 ± 9.06
**BMI**	29.3 ± 4.5
**Sex (M:F)**	5:15
**Disease duration (years)**	11.53 ± 10.22 (median: 6, IQR: 12)
**CRP (mg/l)**	
**○Normal**	15 (75%)
**○High**	2 (10%)
**Employment**	
**○Yes**	47.6%
**○No**	52.4%
**History of foot and ankle injury**	
**○Yes**	57%
**○No**	43%
**DMARDs**	5 (25%)
**Biological**	6 (30%)
**DMARDs and biological**	7 (35%)
**DMARD and/or biological therapy**	18 (90%)
**Change in medication during the study**	2 (10%)
**Foot type**	
**○Normal foot**	5 (25%)
**○Pes cavus**	8 (40%)
**○Pes planus**	7 (35%)
**Deformities**	
**○Rearfoot valgus**	13 (65%)
**○Hallux valgus**	5 (25%)
**○Hallux rigidus**	5 (25%)
**○Hammer toes**	14 (70%)
**Pain site**	
**○Toes**	16 (80%)
**○Metatarsus**	16 (80%)
**○Heel**	11 (55%)
**○Ankle**	17 (85%)

Values are mean ± standard deviation and percentages for categorial variables. *BMI* body mass index, *M* males, *F* females, *CRP* C-reactive protein, *DMARD* disease-modifying antirheumatic drug

### Foot function and global and lower limb pain

**Fig. 1:**
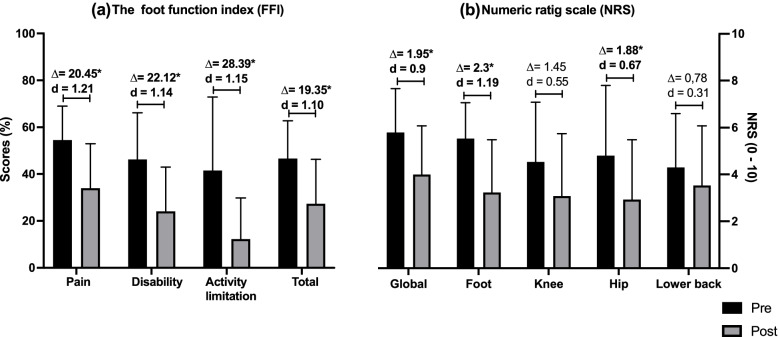
***a*** Foot function and ***b*** global and lower limb pain pre- (baseline) and post-intervention period. *Significant differences (*p* values < 0.05) in mean values between baseline and final follow-up. *FFI* foot function index, *NRS* numerical rating scale, *d* Cohen’s effect size

Figure 1 presents the FFI (Fig. 1a) and NRS (Fig. 1b) scores at baseline and after 7 weeks of using the CFO. All the FFI sub-scores and total score decreased at T7 indicating a significant improvement in foot pain (54.46 ± 14.58% at baseline and 34.01 ± 18.94 at T7), foot disability (46.26 ± 19.91% at baseline and 24.13 ± 18.84 at T7), and foot-related activity limitation (41.54 ± 31.35% at baseline and 12.26 ± 17.57 at T7). The mean pre-post difference in the FFI total score was 22.30 ± 24.76% (*p* = 0.004) and a large effect size (*d* = 1.25) was reported. A significant improvement in foot pain was also demonstrated through the NRS score which decreased by 2.30 ± 2.98 points and a large effect size was reported (*d* = 1.19, *p* = 0.004). Global pain was also significantly decreased with a mean difference of 1.95 ± 2.77 and a large effect size (*d* = 0.90, *p* = 0.006). Regarding lower limb pain, while there were no significant changes in knee and lower back pain, hip pain significantly improved with a mean difference of 1.88 ± 3.38 points and a moderate effect size (*d* = 0.67, 0.031) (Table 2).

**Table 2:** Spatiotemporal parameters pre- (baseline) and post-intervention

Variables	Pre	Post	***p***
	Mean ± SD	Mean ± SD	
**Cadence (step/min)**	108.63 ± 10.87	107.54 ± 9.44	0.126
**Gait cycle duration (s)**	1.12 ± 0.14	1.13 ± 0.11	0.104
**Gait speed (m/s)**	1.09 ± 0.23	1.07 ± 0.18	0.295
**Stride length (m)**	1.19 ± 0.18	1.19 ± 0.13	0.55
**Double support (% GCT)**	21.48 ±3.95	22.25 ± 3.19	*0.014
**Swing time (% GCT)**	39.25 ± 2.00	38.92 ± 1.56	*0.03
**Foot strike angle (degrees)**	25.18 ± 3.91	25.89 ± 3.25	0.279
**Stride time variability (%)**	4.10 ± 3.74	3.89 ± 1.64	0.167

Values are mean ± standard deviation. *SD* standard deviation, *GCT* gait cycle time. *Significant difference (*p* values < 0.05)

**Fig. 2:**
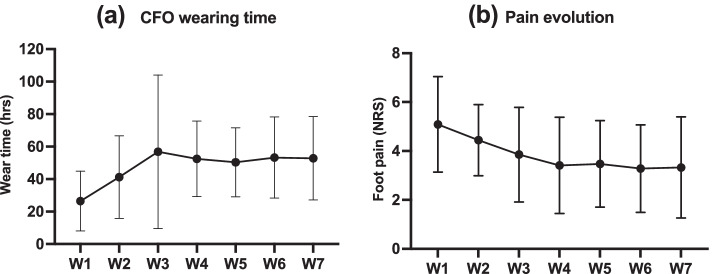
**a** Weekly CFO wearing time and **b** weekly foot pain intensity recorded in the patients’ diaries using the NRS

**Fig. 3:**
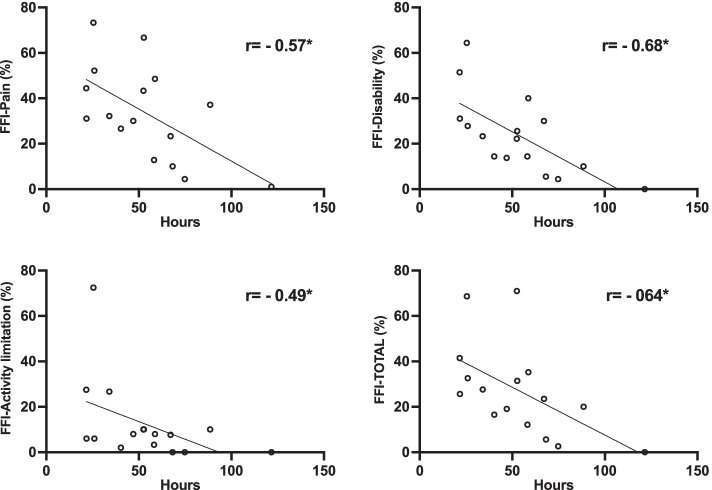
Correlation between weekly CFO wearing time averaged for the last 5 weeks of the intervention period and the FFI subscales and the total score recorded at T7

Seventeen participants recorded the weekly foot pain intensity and CFO wearing time using the provided diaries. The CFO weekly wearing time and the evolution of foot pain are presented in Fig. 2a and b. As it can be observed, there was a tendency for foot pain to decrease during the first 4 weeks and a plateau was observed after this period. The same tendency was also observed for wear time which increased progressively during the first 4 weeks and remained almost stable afterward. The one-way repeated measures ANOVA showed statistically significant changes in weekly foot pain intensity over time *F* (2.13, 32.02) = 20.28, *p* = 0.001, and CFO wearing time *F* (2.27, 34.17) = 5.92, *p* = 0.005. Post hoc tests (with Bonferroni adjustment) showed no significant differences in foot pain between the different time points. However, the differences between week 1 and week 7 (mean difference = 1.81, *p* = 0.09) and weeks 2 and 7 (mean difference = 1.43, *p* = 0.06) were almost significant. Regarding CFO wearing time, post hoc analysis showed a significant decrease between the first week and all the other time points (*p* < 0.05). Correlation coefficients showed significant correlations between the CFO wearing time (hours/week) and foot pain (*r* = −0.57, *p* = 0.023), disability (*r* = −0.68, *p* = 0.005), activity limitation (*r* = −0.49, *p* = 0.047), and the FFI total score (*r* = −0.64, *p* = 0.01) at T7 (Fig. 3).

### Gait function

Spatiotemporal gait parameters measured before and after the intervention period are presented in Table 2. Results show a slight but significant increase and decrease in double support time and swing time, respectively, at T7. However, for all the remaining STP including cadence, gait cycle duration, gait speed, stride length, and foot strike angle, there were no significant differences at T7 compared to baseline. Similarly, no significant changes were reported for the effects of CFO on stride time variability.

### Free-living walking activities (FWA)

**Fig. 4:**
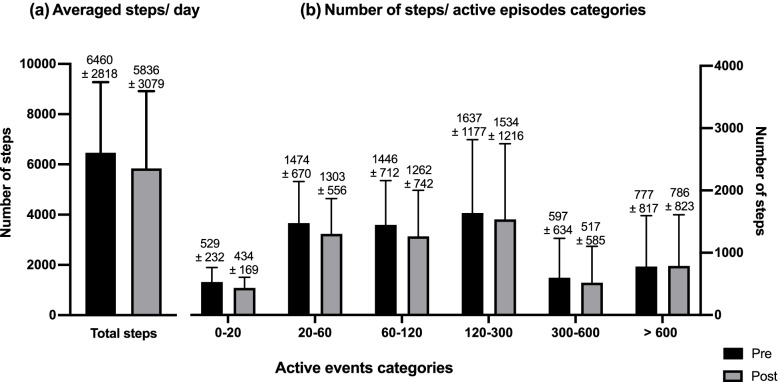
Step count. **a** Daily averaged steps and **b** number of steps of the different step-based active episode categories

**Fig. 5:**
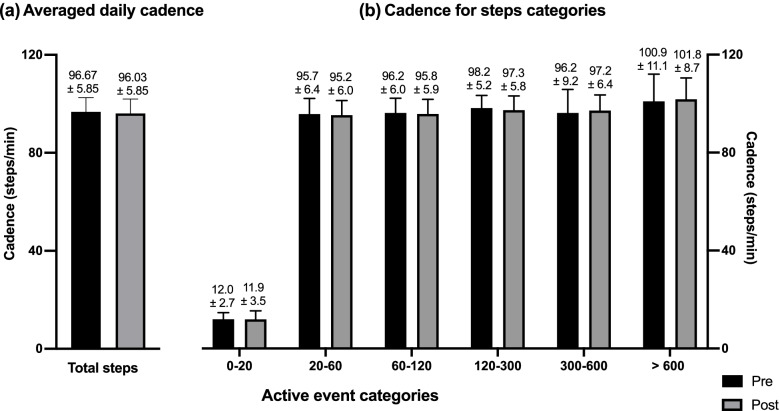
Free-living cadence. **a** Free-living daily averaged cadence and **b** cadence for the different step-based active event categories

**Fig. 6:**
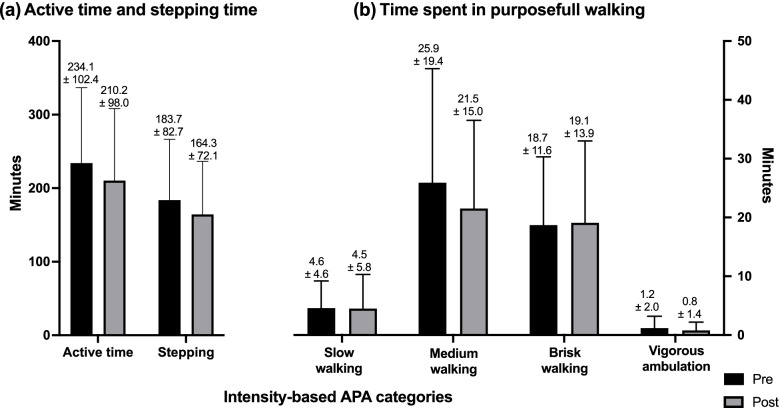
Time spent in APA intensity-based categories. **a** Total active time and time spent in stepping activities and **b** time spent in purposeful walking including only active events of more than 60 steps

Free-living walking activities are presented in Figs. 4, 5, and 6. Out of the 20 participants, four were excluded from the FWA analyses because they did not accumulate at least one valid day of recordings (i.e., recording time > 8 h) at baseline and T7. Consequently, the results presented in this section include data from 16 participants. On average, the patients accumulated a total of 10.79 ± 1.18 h and 9.99 ± 1.02 h of recorded time at baseline and T7, respectively, with no significant differences between baseline and T7 (*p* = 0.09). The average active time was 3.9 ± 1.7 h at baseline and 3.5 ± 1.6 h at T7 (*p* = 0.08). The average number of steps per day was 6460 ± 2818 steps/day at baseline and 5836 ± 3079 steps/day at the end of the intervention period with no significant differences between the two time points (*p* = 0.18) (Fig. 4a). The pattern of accumulating these steps is presented in Fig. 4b. As can be observed, the total number of steps was accumulated in short active events composed of 20 to 300 consecutive steps (Fig. 4b). The daily average free-living cadence was 96.68 ± 5.85 steps/min and 96.03 ± 5.58 steps/min before and after the intervention, respectively (*p* = 0.79) (Fig. 5a). Results show that cadence increases considerably for active events of more than 20 steps compared to those of less than 20 steps (Fig. 5b). For active events composed of 20 to 600 consecutive steps, the mean cadence was 96.65 ± 1.10 steps/min at baseline and 96.38 ± 1.02 steps/min at T7. Results also show that cadence reaches and crosses 100 step/min only for active events composed of more than 600 consecutive steps, for an average of 100.94 ± 11.06 steps/min at baseline and 101.75 ± 8.73 steps/min at T7 with no significant differences between the two time points (*p* = 0.44).

Figure 6 presents minutes spent in APA intensity-based categories including stepping activities (Fig. 6a) and slow, medium, brisk walking, and vigorous APA (Fig. 6b). Out of 234.1 min of active time recorded at baseline, 183.7 min (78 %) were spent in stepping activities and 50.4 min (22 %) were spent in purposeful walking. This same pattern was observed at T7: out of 210.2 min of active time, 164.3 min (78 %) were spent in stepping activities and 45.0 min (22%) spent purposeful walking with no significant differences between the two time points. Participants accumulated 25.9 ± 19.4 min and 18.7 ± 11.6 min of medium and brisk walking at baseline and 21.5 ± 15.0 19 min and 19.1 ± 13.9 min at T7. Time spent in vigorous APA was 1.2 ± 2.0 min at baseline and 0.8 ± 1.4 min at the final follow-up (*p* = 0.52).

## Discussion

The primary purpose of this study was to explore the effects of wearing CFO for a 7-week period on foot function in people with PsA. As a secondary objective, we sought to assess the effects of CFO on foot pain, gait function (instrumented gait STP analysis), and FWA and to assess the relationship between the CFO wearing time and foot pain and function in people with PsA with foot involvement.

### Foot pain and foot function

The main findings showed a significant improvement in foot pain (NRS and FFI) and foot function (FFI) after using the CFO for a 7-week intervention period. The FFI total score decreased by 22.30%, and a large effect size (*d* = 1.25) was reported. Interestingly, after only 7 weeks of intervention, this improvement was clinically significant for 70% of the participants since the pre-post differences in their FFI total scores were higher than the MCID previously reported for the FFI total score [49].

Research in people with RA has shown some efficacy of CFO on foot pain which is consistent with our findings [41–43]. However, conflicting results regarding the effects of CFO on foot function have been reported. Indeed, while some studies endorsed the effectiveness of CFO on foot function [44, 46], others reported no significant effects for this outcome [58–60]. Several factors could explain such discrepancies. First, the orthoses type can differ between the studies according to the targeted therapeutical aim (e.g., palliative vs functional). Moreover, foot casting and manufacturing materials and techniques can vary from one study to another depending on the latest advances in technologies and on the national practice guidelines making between-studies comparison difficult. In our study, we used *functional CFO* in all the participants. The materials and techniques of foot casting and manufacturing were standardized for all the participants. However, the degrees and types of corrections were specific for each participant’s needs and tolerance. The CFO were also adjusted at delivery and during the following 2 weeks to increase comfort and acceptance if needed. This could explain the positive effects on pain and foot function observed in this study. Furthermore, there was no consensus regarding the previous studies’ intervention durations and control conditions. Some trials used placebo orthoses while others used shoes only as a control condition. As the former are usually made from thick and cushioned materials, they cannot be considered as not having therapeutic effects, thereby attenuating the CFO effects. In contrast, using shoes-only as a control could overestimate the effect of CFO [61].

Importantly, the CFO wearing time was not documented in all the previous studies which can compromise their conclusions as a lack of effectiveness could partially be related to insufficient use of the orthoses. In our study, a sufficient wearing time (8h/day) was reported and a strong correlation between the CFO wearing time and the FFI total score was demonstrated. These findings are similar to those reported in a few studies in people with RA that did report wearing time [44, 46, 60]. This observation highlights the relevance of monitoring this variable in future studies and the importance of continuous and regular wearing of the CFO.

Apart from the intervention characteristics, sampling groups and individual characteristics such as age, sex distribution, disease duration, and baseline levels of foot pain and disability are also important considerations as they may influence the effectiveness of CFO. For instance, data from a previous study [62] showed that shorter disease duration, younger age, and higher baseline values of foot pain and disability predicted improved outcomes after CFO use. In the present study, the participants had a mean age of 54.10 ± 9.06, a median disease duration of 6 years, a high prevalence of foot pain, and severe levels of disability at baseline. Therefore, it is not unexpected that the CFO helped relieve pain and improve perceived foot function. In addition to foot pain and disability, the findings showed a significant decrease in global and hip pain. However, the differences were not clinically significant (mean differences < 2 points). Knee and lower back pain also improved but the differences did not reach the significance level. Although there is evidence that CFO could have beneficial effects on lower limb pain [63–66], further studies using specific tools to assess knee, hip, and lower back pain in detail are needed to confirm the beneficial effects of CFO on these outcomes.

### Gait function

Gait STPs are indicators of functional capacity and overall health and autonomy, making them the most relevant and most often measured biomechanical parameters for gait analysis in healthy and pathological populations [13, 67]. We noticed a lack of scientific evidence regarding the effects of CFO on gait STPs in people with arthritic foot diseases. For instance, studies in people with RA showed only a minimal or no effect of CFO for increasing gait speed, cadence, and stride length [42, 68, 69] and some studies showed that wearing the CFO allowed for an improvement of gait stability [45, 70].

Our findings showed that gait STPs were altered compared to the normative values reported for healthy adults [71, 72]. However, there were no significant changes in STP and gait stability after the intervention except for a negligible effect on double support and swing time. These results could be potentially attributed to the short-term nature of our intervention period and the long disease duration reported in this study. Gait impairments demonstrated in participants with PsA may be acquired over many years. Consequently, the patients can develop antalgic walking strategies that may require more extended intervention periods to improve. In addition, the patients could have developed fear-avoidance beliefs which have been reported as significant predictors of poorer response to CFO in other patient populations [73]. Another possible explanation for the lack of STP response could be that PsA may be more responsive to CFO treatment in earlier stages of the disease. Therefore, long-term studies including patients with early disease would be more relevant to assess the effects of CFO on gait STP properly.

Furthermore, although the 10MWT is reliable and responsive in patients with major gait impairments secondary to neurological diseases such as stroke and Parkinson [74], it might not have been responsive in this study because of the relatively milder gait impairment related to PsA and the short intervention’s duration and follow-up period [75]. Longer time/distance tests should also be considered when assessing gait in people with PsA as gait impairments reported in this population could be attributed to common features of inflammatory arthritis such as muscle weakness, reduced range of motion, and deformities [76–78] and they may be more significant during sustained walking activities.

### Free-living walking activities

The benefits of physical activity on health are undeniable. Results from a recent systematic review [26] suggest that physical activity may positively affect disease activity, muscle strength, fatigue, pain, quality of life, and cardiovascular risk in people with PsA. Therefore, promoting physical activity in this population should be a health care priority. Walking is a practical, accessible, and low-skill mode of physical activity that plays a major role in occupational and social activities of everyday living [79]. The findings of this study reported decreased step counts with large standard deviations (6460 steps/day at baseline and 5836 steps/day at T7) which are below the recommended 10,000 steps/day [80]. Similarly, the daily averaged free-living cadence was 96.67 ± 5.85 steps/min at baseline and 96.03 ± 5.85 steps/min at T7 which is below the naturally selected free-living walking speed (100 steps/min) reported for healthy individuals [56, 81]. Besides, the reported time spent in brisk walking did not achieve the minimum of daily 30 min of moderate-intensity physical activity recommended by the international guidelines for PA activity [82]. These results indicate decreased PA levels in participants with PsA with foot problems which is consistent with what is reported in a previous systematic review [26]. Future studies comparing PA levels between participants with PsA and matched healthy controls are needed to confirm these results.

We postulated that by alleviating pain, the use of CFO would positively impact walking activities in people with PsA. Nevertheless, neither the number of steps/day, free-living cadence, nor the time spent in the different intensity-based APA changed after using the CFO. Again, the short-term nature of this study could have not fully reflected the effects of CFO. Besides, free-living cadence is a measure of ambulatory behavior [56] that is likely influenced by psychoaffective factors that have not been assessed in this study. In a previous study in people with PsA, beliefs about PA (e.g., fear of exacerbating pain) have been shown to be associated with activity level [83]. This suggests that future studies should address the patient’s behavior which is complex and may require using a multidisciplinary approach including behavioral change strategies, advice, education, prescription of exercise therapy, and CFO to promote physical activity in people with PsA. In addition, as this study was carried out over nearly 2 years, natural environmental factors such as seasons/weather, and the occurrence of the COVID-19 pandemic, might have influenced the patients’ PA [84, 85].

Lastly, it should be mentioned that only 16 participants were included in the FWA analyses and there might be not enough power to detect significant changes. Also, the average recorded time was nearly 1 h lower at T7 compared to baseline (10.79 h vs 9.99). This could have contributed to the obtained results. To our knowledge, only one study investigated the effects of CFO on physical activity [86] in a population of people with RA. Using a self-reported questionnaire, their results showed that a 6-month CFO use improved light-intensity physical activity but not moderate- and vigorous-intensity activities [86]. However, these findings cannot be compared to those demonstrated in the present study because of the different approaches used for PA measurement since using self-reported measures could overestimate PA compared to objective measurements [87].

This study has limitations that should be mentioned. First, there was no control group to control for the placebo effect and the natural evolution of the disease. Future studies are encouraged to use prefabricated, sham orthoses and/or a shoe-only condition as comparators to overcome this limitation. The choice of the control condition should be made according to the study aims (e.g., using prefabricated orthoses as a control condition if the aim is to assess the intervention’s cost-effectiveness). Moreover, if sham orthoses are to be used, researchers are encouraged to document the credibility and the biomechanical effects of these devices. Second, the follow-up duration may have been too short to show changes in outcomes such as gait parameters and FWA that may necessitate a longer time to improve. Moreover, the small sample size and the high proportion of female participants which is not representative of the gender distribution usually seen in PsA could compromise the generalizability of our findings.

## Conclusion

The use of CFO for a 7-week period improved foot pain and related disability in people with PsA. This suggests that CFO may be used in conjunction with pharmacological therapy to relieve pain and improve perceived foot function. However, the results should be confirmed in larger and controlled studies. The effects of CFO on gait function and free-living walking activities were not evident in this study due to the small sample size, short follow-up period, and personal and environmental factors that have not been measured. Future RCTs with larger sample sizes and longer intervention durations are needed for any conclusions to be drawn.

## Supplementary Information

**Additional file 1.** Signal processing and steps identification.

**Additional file 2.** Flow diagram.

## Data Availability

The dataset used and analyzed during the current study is available from walha.roua@usherbrooke.ca on reasonable request.

## Abbreviations

APA: Ambulatory physical activity
BMI: Body mass index
CRP: C-reactive protein
CFO: Custom foot orthoses
DMARD: Disease-modifying antirheumatic drug
FFI: Foot function index
FWA: Free-living walking activities
GCT: Gait cycle time
MCID: Minimal clinically important difference
NRC: Numerical rating scale
PA: Physical activity
PsA: Psoriatic arthritis
STPs: Spatiotemporal parameters
10MWT: 10-m walk test
