# Supplementary material for: The effects of custom-made foot orthoses on foot pain, foot function, gait function, and free-living walking activities in people with psoriatic arthritis (PsA): a pre-experimental trial
Source: Arthritis Res Ther. 2022 May 25;24:124. doi: 10.1186/s13075-022-02808-8 (PMC9130455; doi:10.1186/s13075-022-02808-8)
Supplement: Supplementary file 1 — Additional file 1. Signal processing and steps identification. [file 13075_2022_2808_MOESM1_ESM.docx]

**Additional file**
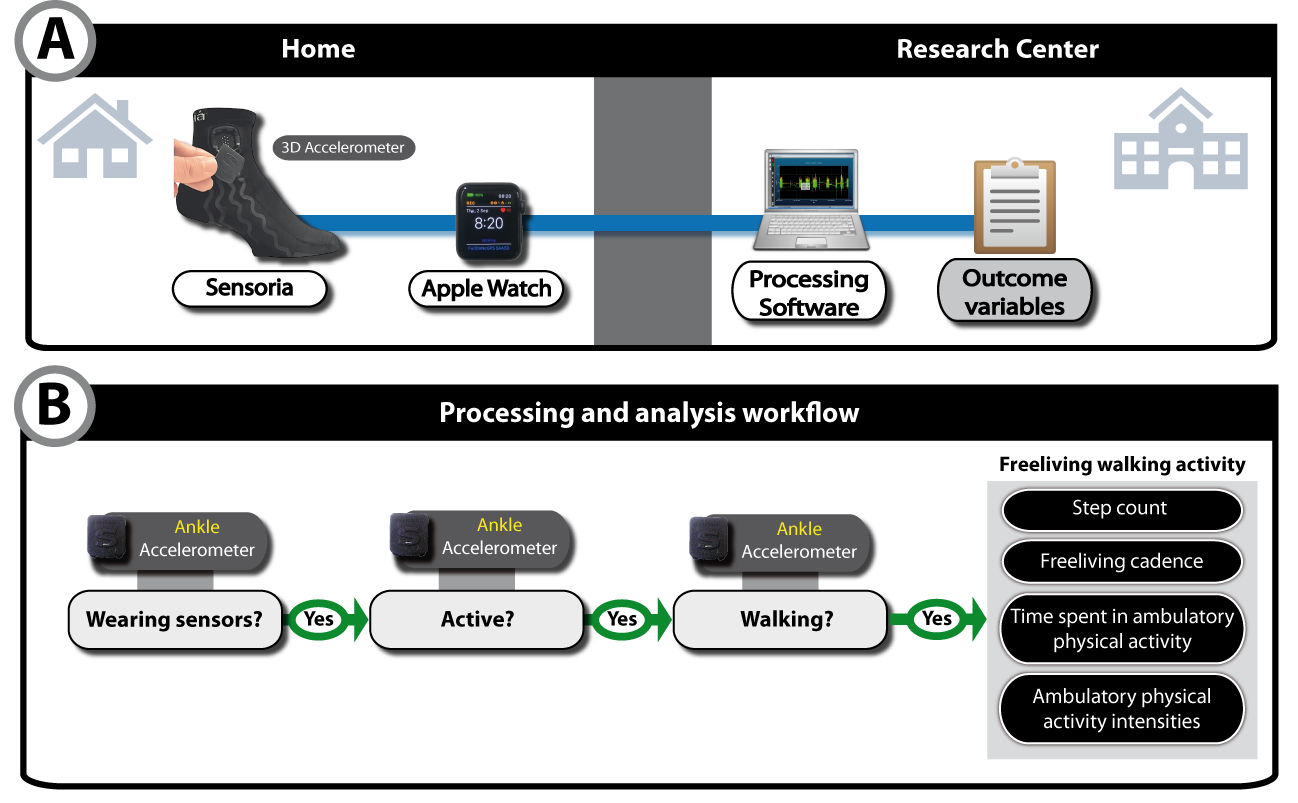
**: Signal processing and Steps identification:**

Briefly, the steps detection was based on the norm of the acceleration signal extracted from the instrumented sock attached-IMU. First, motion information was recovered by filtering the low frequency content of the signal using a 2nd order high pass Butterworth filter and a zero-phase filter. Active and inactive intervals were then determined by applying the Euclidean norm twice to the vector which was then filtered using a 2^nd^ order low pass Butterworth filter, compared to a certain threshold, and finally normalized between 0 and 1. The resulting vector reflects the density of active time. Hence, each point above 0.5 is considered active otherwise it is considered inactive. An interval of active point was defined as a succession of points considered as active with a tolerance of 10 seconds of consecutive inactive points. Steps were identified and counted from the active intervals by first computing the Fast Fourier transform (FFT) function of the raw accelerometer signal to discriminate the walking speed. Afterwards, an aggressive low pass filter was applied to keep only walking data. An envelope detection and removal were then performed to discriminate peaks and a moving average was applied to identify the peaks on this modified signal. Each peak is related to a step. Once steps have been detected, walking events were then determined as every interval of the signal where at least 5 consecutive steps were detected. Walking time was calculated as the sum of time spent in walking events. Cadence was then computed for each walking event and for the total walking time/day. Based on cadence data, the sum of minutes spent in 0 to 60 steps/min, 60 to 79 step/min, 80 to 99 step/min, 100 to 119 step/min and > 120 step/min was calculated for purposeful walking defined as all active events (i.e. walking events) composed of more than 60 consecutive steps.
