# Supplementary material for: The effects of custom-made foot orthoses on foot pain, foot function, gait function, and free-living walking activities in people with psoriatic arthritis (PsA): a pre-experimental trial
Source: Arthritis Res Ther. 2022 May 25;24:124. doi: 10.1186/s13075-022-02808-8 (PMC9130455; doi:10.1186/s13075-022-02808-8)
Supplement: Supplementary file 2 — Additional file 2. Flow diagram. [file 13075_2022_2808_MOESM2_ESM.doc]

**Additional file 2: Flow Diagram**

**Allocation**

**Analysis**

**Follow-Up**

**Enrollment**

Assessed for eligibility (n=27)

Excluded (n= 5)

♦  Not meeting inclusion criteria (n= 2)

♦  Declined to participate (n= 3)

Lost to follow-up (n= 0)

Discontinued intervention (acute outbreak and hospitalization due to acute arthritis flare) (n= 1)

Allocated to intervention (n= 22)

♦ Received allocated intervention (n= 21)

♦ Did not receive allocated intervention (participant had plantar fibromatosis that required surgery) (n= 1)

Analysed (n= 20)
♦ Excluded from analysis (n=0)

No randomization
